# Association of metabolic obesity phenotypes with cognitive decline in the ELSA‐Brasil study

**DOI:** 10.1111/dom.70466

**Published:** 2026-01-20

**Authors:** Paulo Henrique Lazzaris Coelho, Natalia Gomes Gonçalves, Itamar S. Santos, Alessandra C. Goulart, Sandhi Maria Barreto, Luana Giatti, Paulo Caramelli, Paulo Andrade Lotufo, Isabela Martins Bensenor, Claudia Kimie Suemoto

**Affiliations:** ^1^ Division of Geriatrics, University of São Paulo Medical School São Paulo São Paulo Brazil; ^2^ Preventive Medicine Department University of São Paulo Medical School São Paulo São Paulo Brazil; ^3^ Center for Clinical and Epidemiological Research, Hospital Universitário, University of São Paulo São Paulo São Paulo Brazil; ^4^ Department of Preventive and Social Medicine & Clinical Hospital/EBSERH Universidade Federal de Minas Gerais Belo Horizonte Minas Gerais Brazil; ^5^ Behavioral and Cognitive Neurology Unit, Faculdade de Medicina, Universidade Federal de Minas Gerais Belo Horizonte Minas Gerais Brazil

**Keywords:** cohort study, elderly, macrovascular disease, obesity care, observational study, weight management

## Abstract

**Aims:**

To investigate the association between metabolic obesity phenotypes and cognitive decline and evaluate the potential mediating role of C‐reactive protein (CRP).

**Methods:**

Longitudinal cohort study using three waves (2008–2019) of the Brazilian Longitudinal Study of Adult Health (ELSA‐Brasil). Data were analysed from December 2024 to May 2025. Baseline sample consisted of 15 105 participants aged 35–74 years. Six phenotypes were defined by body mass index (BMI) category (normal weight, overweight, obesity) and metabolic health. Metabolic health was defined using traditional metabolic syndrome criteria and more stringent criteria: low waist‐to‐hip ratio (WHR), no diabetes or hypertension. Global cognition Z‐scores were derived from tests of memory (immediate, delayed recall, and recognition of a word list), verbal fluency (phonemic and semantic), and Trail‐Making tests (TMT‐B). Mediation analysis evaluated CRP as a potential mediator.

**Results:**

Among 12 795 participants (mean age 51.1 years; 55% women; 53% White) followed for a median of 8 years, metabolically unhealthy phenotypes—across all BMI categories—were associated with faster cognitive decline (β estimates ranged from −0.037 to −0.053; all *p* < 0.001), whereas metabolically healthy overweight (β = 0.016; 95% CI = −0.002, 0.034; *p* = 0.081) and metabolically healthy obesity (β = 0.000; 95% CI = −0.027, 0.026; *p* = 0.981) were not. No evidence of CRP mediation was identified. BMI was not associated with cognitive decline (β = −0.001; 95% CI = −0.002, 0.000; *p* = 0.170), whereas WHR was (β = −0.020, 95% CI = −0.026, −0.014, *p* < 0.001).

**Conclusions:**

Metabolic dysfunction may be a stronger predictor of subsequent cognitive decline than excess body weight. Dementia prevention strategies may benefit from early identification and management of metabolic dysfunction across all weight categories.

## 1 INTRODUCTION

Midlife obesity is a well‐established risk factor for cognitive decline and dementia, yet evidence increasingly suggests that obesity is a heterogeneous condition and that body mass index (BMI) alone may misclassify health risks.^1^, ^2^, ^3^, ^4^, ^5^, ^6^, ^7^, ^8^, ^9^, ^10^ Recent literature has highlighted the concept of metabolic obesity phenotypes, which differentiate individuals with similar BMI based on their metabolic profiles.^11^ In this context, the notion of metabolically healthy obesity (MHO) has emerged, describing individuals with obesity‐range BMI who do not exhibit typical metabolic abnormalities.^12^ Consequently, these individuals have a lower risk of adverse health outcomes than their metabolically unhealthy counterparts.^11^, ^12^ These observations raise the question of whether metabolic dysfunction, rather than excess body weight per se, is the feature most strongly associated with subsequent cognitive decline.

Much of the research about metabolic obesity phenotypes has focused on cardiovascular outcomes, and studies on cognitive outcomes remain scarce.^6^, ^13^, ^14^, ^15^, ^16^ Most prior study has evaluated incident dementia in North‐American populations or cognitive decline in older Asian populations and has operationalized MHO using the National Cholesterol Education Program Adult Treatment Panel III (NCEP‐ATP III) criteria, which classify individuals with one metabolic risk factor as metabolically healthy.^14^, ^17^, ^18^ Recently, a stricter definition has been proposed, identifying MHO as obesity‐range BMI accompanied by a low waist‐to‐hip ratio (WHR) and absence of hypertension or diabetes.^12^ Under this newer definition, MHO has been associated with a low risk of cardiovascular outcomes, in contrast to the intermediate risk observed with the traditional NCEP‐ATP III criteria.^12^ Because these definitions potentially capture different metabolic profiles, they may also yield different associations with cognitive outcomes, yet this has been minimally explored.

Additionally, given the links between metabolic dysfunction and neurodegeneration, inflammation has been proposed as a potential pathway.^19^ In particular, low‐grade systemic inflammation measured by C‐reactive protein (CRP) is associated with metabolic syndrome and neurodegeneration and may mediate the relationship between metabolic health and cognition.^20^, ^21^

We primarily investigated whether baseline metabolic obesity phenotypes were associated with cognitive decline over follow‐up. Secondarily, we examined whether baseline CRP mediated these associations and explored the associations of WHR and BMI with cognitive decline. We aimed to address key limitations of prior studies by applying a more stringent and contemporary definition of metabolic health and by leveraging data from a large, ethnically diverse Brazilian cohort with a prospective design beginning in midlife, which is a period increasingly recognized as critical for shaping cognitive trajectories.^22^

## 2 METHODS

### 2.1 Study design

The Brazilian Longitudinal Study of Adult Health (ELSA‐Brasil) is a multicenter prospective cohort study designed to investigate the incidence of chronic diseases, particularly cardiovascular diseases and diabetes, and their risk factors in the Brazilian population.^23^ The main baseline exposures include sociodemographic characteristics, lifestyle factors, cardiometabolic risk factors, and subclinical disease markers, while the primary outcomes comprise cardiovascular events, diabetes, and other chronic conditions.^23^ The cohort comprises civil servants from public universities and a research institute across the capitals of six Brazilian states (Bahia, Minas Gerais, São Paulo, Espírito Santo, Rio de Janeiro, and Rio Grande do Sul). Data were collected in three waves, approximately four years apart (2008–2010, 2012–2014, and 2017–2019).^23^, ^24^ Participants provided informed consent before enrollment. The ELSA‐Brasil study was approved by the Ethics and Research Committees of all institutions participating in the study and registered at the National Research Ethics Committee (letter 976 CONEP/CNS/MS, on August 4th/2006).

In this secondary analysis of the ELSA‐Brasil cohort, we investigated the longitudinal association between baseline metabolic obesity phenotypes and cognitive decline.

### 2.2 Participants

**FIGURE 1:**
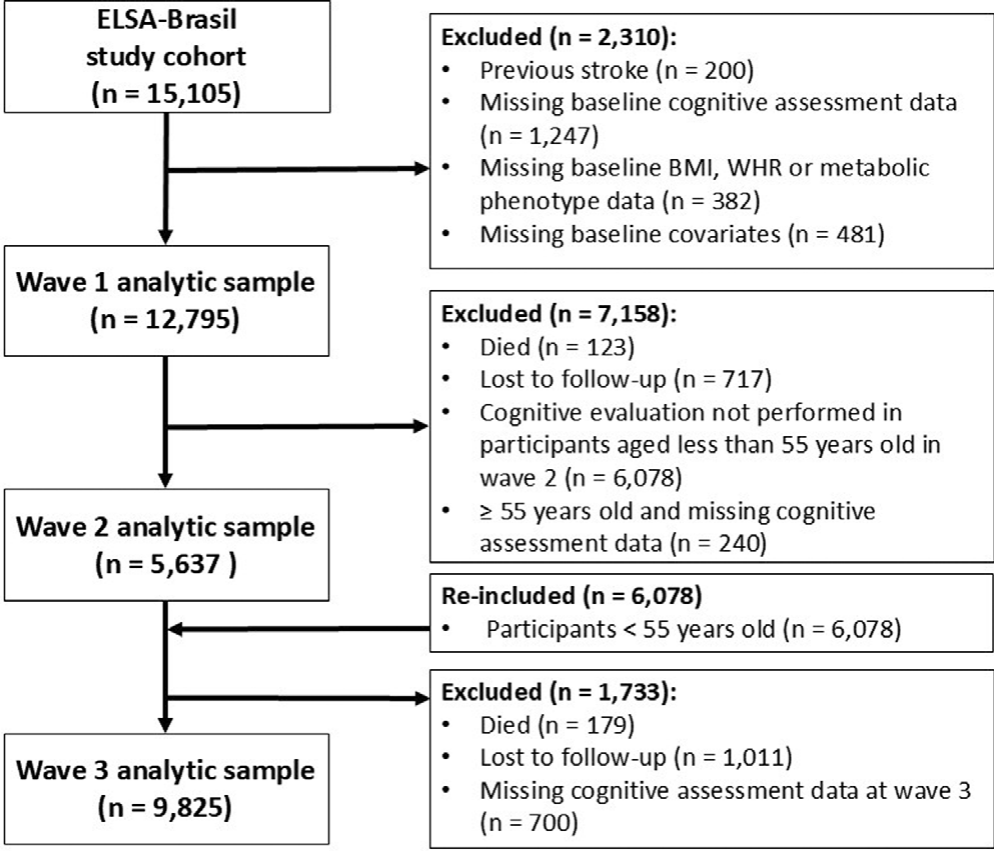
Flow diagram for study participants.

ELSA‐Brasil recruited active and retired public servants, excluding individuals with cognitive or communication impairments, those intending to leave their institution for reasons other than retirement, and retirees residing outside metropolitan areas. The baseline sample consisted of 15 105 participants aged 35–74 years. For this analysis, we sequentially excluded participants with a history of stroke (*n* = 200), missing baseline cognitive assessment data (*n* = 1247), missing baseline BMI, WHR, or metabolic phenotype (*n* = 382), underweight participants (*n* = 134), and those missing baseline covariates (*n* = 481). Underweight (BMI <18.5 kg/m^2^) participants were excluded due to their small sample size (*n* = 134), distinct health risks, and the potential for reverse causation bias, as underweight status in midlife may reflect underlying chronic disease or early manifestations of cognitive decline.^25^ Participants missing cognitive assessments in later waves, deceased, or lost to follow‐up were removed only from the respective waves' analytic sample. Additionally, due to the ELSA‐Brasil study design, participants younger than 55 years were not assessed for cognitive function in the second wave, which led to their exclusion from that wave's analytic sample; however, they were reincluded in the third wave (Figure 1).

### 2.3 Metabolic obesity phenotypes classification

Metabolic obesity phenotypes were defined based on BMI categories and metabolic health status. BMI was categorized as normal weight (18.5–24.9 kg/m^2^), overweight (25.0–29.9 kg/m^2^), and obesity (≥30.0 kg/m^2^). Participants were classified as metabolically healthy if they met all of the following criteria: (1) absence of diabetes, (2) systolic blood pressure <130 mmHg and no antihypertensive medication, and (3) WHR below sex‐specific thresholds (<0.95 for women and <1.03 for men).^12^ Those who did not meet any of these criteria were classified as metabolically unhealthy. This definition, which reflects a stricter and more recent approach to metabolic health, was used as the primary classification for the main analyses.^12^ Based on this definition, participants with obesity were classified as MHO or metabolically unhealthy obesity (MUHO). Similarly, overweight and normal‐weight individuals were categorized as metabolically healthy overweight (MHOW), metabolically unhealthy overweight (MUHOW), metabolically healthy normal‐weight (MHNW), and metabolically unhealthy normal‐weight (MUHNW). This definition was prioritized as the main exposure because it reflects the most contemporary conceptualization of metabolic health and required fewer variables, resulting in substantially less missing data.

Participants were classified into the same six phenotype categories (MHNW, MHOW, MHO, MUHNW, MUOW, MUO) using the NCEP‐ATP III criteria for metabolic health. This alternative classification was included to ensure comparability with prior epidemiological studies and to evaluate the robustness of the main findings across alternative operational definitions. A detailed description of the NCEP‐ATP III criteria, diabetes definition, and anthropometric measurements is provided in the Data S1. We compared both definitions classifications using complete‐cases cross‐tabulation and unweighted Cohen's κ (Table S1). Agreement was substantial (κ = 0.64; 95% CI: 0.63–0.65), yet 29% of participants were reclassified across definitions, which motivated the use of this alternative definition in sensitivity analyses.

### 2.4 Cognitive assessment

Cognitive function was evaluated using six standardized tests covering memory, language, and executive function.^26^ Memory was assessed through the immediate recall, delayed recall, and recognition components of the Consortium to Establish a Registry for Alzheimer's Disease (CERAD) word list memory test.^27^ Language was assessed using a semantic verbal fluency test (animal categories in the first and third waves, vegetables in the second wave) and a phonemic verbal fluency test (letter “F” in the first and third waves, letter “A” in the second wave).^28^ Verbal fluency test results were harmonized across waves to account for the different categories and letters used.^29^, ^30^ Executive function was assessed using the Trail‐Making Test part B (TMT‐B).^31^ Z‐scores were calculated for each test relative to wave 1. The TMT‐B Z‐score was multiplied by −1 because higher scores in this test denote poorer performance, while higher scores in the other tests indicate better performance. A global cognition Z‐score standardized to the first wave was obtained as the arithmetic mean of the Z‐scores of the six tests, with equal weighting across cognitive domains. Further details on the cognitive assessments and standardization procedures are available in the Data S1.

### 2.5 Covariates

Potential sociodemographic, clinical, and lifestyle confounders in the association between metabolic phenotypes and cognitive decline were selected as covariates. Data on age, sex (male/female), race, marital status, education level, smoking status, and alcohol use were obtained using standardized questionnaires.^32^ Given the small proportion of participants with less than high school education (<10%), the education variable was dichotomized into “high school or less” and “college or more”. Individuals who were formally married or in a stable relationship were classified as married. Race was self‐reported and categorized based on the Brazilian Institute of Geography and Statistics (IBGE) classifications used in the national census: Black, Brown (“pardo”), White, Yellow, and Indigenous. To increase statistical power and account for shared socioeconomic and historical factors, Black and Brown participants were combined into a single “Black/Mixed” category, given their similar socioeconomic backgrounds, health outcomes, and experiences of discrimination.^33^, ^34^, ^35^ Indigenous (0.9%, representing Native Brazilians) and Yellow (2.5%, primarily of East Asian descent) participants were grouped as “Other” due to their low frequency in the sample.

Depressive symptoms were measured using the Brazilian version of the Clinical Interview Scheduled Revised (CIS‐R).^36^ Physical activity was assessed with the long‐form International Physical Activity Questionnaire (IPAQ), and physical inactivity was defined as fewer than 150 min of moderate exercise per week or fewer than 75 min of vigorous exercise per week.^37^

### 2.6 Statistical analysis

Descriptive analyses were presented as means and standard deviations (SD) for continuous variables and absolute and relative frequencies for categorical variables. To investigate the association between metabolic phenotypes and cognitive decline, we employed linear mixed models (LMM) with random intercepts and slopes. The term for time used in the model was age at each wave divided by eight. This linear rescaling was performed to improve interpretability.^3^ Consequently, each β coefficient represents the mean change in global cognition Z‐score over an eight‐year period (the study's median follow‐up), with negative values indicating a faster rate of cognitive decline. The primary analyses used MHNW as the reference category. Supplementary analyses used MHO and MUHO as alternative reference categories to explore additional contrasts. The first model was adjusted for baseline age, sex, race, marital status, and education level. The second and final model was further adjusted for baseline alcohol consumption, smoking status, physical inactivity, and depressive symptoms. The same modelling approach was applied using BMI and WHR as continuous exposures instead of metabolic phenotypes. For these exposures, a third model adjusted for hypertension, diabetes, dyslipidemia, and cardiovascular disease, although it must be interpreted cautiously as these variables are potential mediators in the association of BMI and WHR with cognitive decline. These covariates were not included in metabolic phenotype models to avoid overadjustment, as they are part of the definition of metabolic health. WHR was standardized by scaling to its SD to improve numerical stability, hence the effect estimates correspond to a one‐SD increase in WHR. Inverse Probability of Attrition Weighting (IPAW) was applied to address potential attrition bias and is described in greater detail in the Data S1.^38^ Weights were stabilized using baseline covariates and truncated at the 1st and 99th percentiles to minimize the influence of extreme values. The final stabilized weights ranged from 0.40 to 2.58.

Additionally, we investigated whether the associations between metabolic phenotypes and cognitive decline were modified by sex (men/women), race (white and black/mixed, excluding the “other race” category), or older age status (≥60 years) by including three‐way interaction terms between these variables, the timescale, and cognitive performance. When a significant interaction was detected (*p* < 0.05), we stratified the analysis by the sociodemographic variable.

Mediation of the association between metabolic phenotypes and cognitive decline by baseline CRP levels was evaluated using the difference‐of‐coefficients method.^39^ Two LMMs were fitted: the total effect model (i.e., the final model described above) and the direct effect model, which additionally included baseline CRP levels and its interaction with the timescale. The coefficients for the exposure–timescale interaction in these models represent the total and direct effects, respectively, and the indirect effect was computed as their difference. The proportion mediated was calculated as the indirect effect divided by the total effect. Confidence intervals were obtained via 1000 bootstrap iterations. This mediation analysis assumes that: (1) exposure, mediator, and outcome occur in a temporal sequence; (2) there are no unmeasured mediator‐outcome confounders; and (3) there is no mediator‐exposure interaction.

Sensitivity analyses were performed to address the potential bias from excluding participants under 55 years old at the second wave and are described in the Data S1. We applied the last observation carried backward method, imputing cognitive Z‐scores using data from the third wave. This approach was considered conservative, since cognitive function generally declines over time, and thus, carrying scores backward minimizes the risk of overestimating cognitive performance in the imputed data.

The alpha level was set at the 5% level in 2‐sided tests. Statistical analyses were performed using R statistical software version 4.3.2 (R Project for Statistical Computing).

## 3 RESULTS

A total of 15 105 individuals participated in the ELSA‐Brasil cohort, and 2310 were excluded from these analyses, resulting in a final sample of 12 795 participants followed for a median of 8 years (IQR: 8–9 years) (Figure 1). Excluded individuals were more likely to have lower educational attainment, be older, non‐White, metabolically unhealthy, and be at extreme categories of BMI (Table S2). Among the included participants, the majority were women (55.1%), White (52.9%), and had at least a college degree (55.4%) (Table 1). The mean (SD) age was 51.5 (8.9) years. Participants classified in metabolically healthy weight categories were younger, predominantly women, more highly educated, had healthier lifestyles, lower CRP levels, and better cognitive performance (Table 1). Within each metabolic phenotype, CRP levels increased progressively across weight categories (Table 1).

**TABLE 1:** Baseline sociodemographic, clinical characteristics, and cognitive assessment by metabolic phenotype (*n* = 12 795).

	Overall (*n* = 12 795)	MHNW (*n* = 3228)	MHOW (*n* = 2566)	MHO (*n* = 853)	MUHNW (*n* = 1518)	MUHOW (*n* = 2588)	MUHO (*n* = 2042)	*p*‐value^a^
Age (years)	51.5 (8.9)	48.4 (8.1)	48.8 (7.9)	47.9 (7.8)	55.4 (9.0)	55.1 (8.6)	53.8 (8.5)	<0.001
Women	55.1%	63.1%	55.8%	68.9%	49.7%	43.2%	55.0%	<0.001
Race								<0.001
White	52.9%	60.7%	55.7%	52.4%	48.4%	48.4%	46.1%	
Black/Brown	43.7%	35.8%	41.1%	45.7%	46.4%	48.0%	50.9%	
Other	3.4%	3.4%	3.2%	1.9%	5.1%	3.6%	3.0%	
College or more	55.4%	65.9%	59.9%	55.2%	52.4%	48.3%	44.5%	<0.001
Married	66.4%	65.1%	68.3%	64.0%	64.4%	70.5%	63.1%	<0.001
Smoking status								<0.001
Never	57.8%	65.5%	59.3%	60.8%	55.3%	50.6%	53.3%	
Former	29.6%	21.4%	29.1%	27.2%	28.1%	36.9%	36.2%	
Current	12.6%	13.1%	11.6%	12.0%	16.7%	12.4%	10.4%	
Alcohol use								<0.001
Never	10.1%	8.4%	10.4%	9.6%	11.5%	9.9%	11.9%	
Former	19.0%	17.2%	17.1%	21.5%	21.3%	18.5%	22.3%	
Current	70.9%	74.4%	72.5%	68.9%	67.3%	71.7%	65.8%	
Physical inactivity	74.9%	67.4%	71.8%	80.0%	74.6%	78.5%	83.9%	<0.001
Depressive symptoms	12.7%	10.9%	12.9%	15.5%	11.8%	11.8%	16.0%	<0.001
BMI	27.0 (4.7)	22.6 (1.6)	27.0 (1.4)	33.0 (3.1)	22.9 (1.5)	27.5 (1.4)	34.1 (3.8)	<0.001
C‐reactive protein (mg/L)	2.8 (4.5)	1.8 (4.0)	2.3 (3.4)	4.0 (4.1)	2.2 (3.6)	3.0 (5.1)	4.8 (5.4)	<0.001
Semantic verbal fluency	18.8 (5.2)	19.6 (5.1)	19.3 (5.3)	19.3 (5.1)	18.0 (5.1)	18.0 (5.0)	18.2 (5.1)	<0.001
Phonemic verbal fluency	12.8 (4.4)	13.6 (4.2)	13.1 (4.3)	12.9 (4.2)	12.4 (4.5)	12.2 (4.4)	12.1 (4.3)	<0.001
Immediate recall	21.3 (3.8)	22.1 (3.6)	21.5 (3.8)	21.6 (3.6)	20.9 (4.1)	20.6 (3.8)	20.8 (3.8)	<0.001
Word recognition	9.6 (0.9)	9.7 (0.8)	9.6 (0.8)	9.6 (0.8)	9.5 (0.9)	9.5 (0.9)	9.5 (0.9)	<0.001
Late recall	7.0 (2.0)	7.5 (1.8)	7.2 (1.9)	7.1 (1.8)	6.8 (2.1)	6.7 (2.0)	6.8 (2.0)	<0.001
Trail making test version B (seconds)	122.4 (85.4)	105.4 (72.8)	108.8 (67.9)	111.1 (87.2)	139.6 (97.5)	138.9 (94.1)	137.2 (92.6)	<0.001

*Note*: Data are presented as mean (SD) or %.

Abbreviations: BMI, body mass index; MHNW, metabolically healthy normal weight; MHOW, metabolically healthy overweight; MHO, metabolically healthy obesity; MUHNW, metabolically unhealthy normal weight; MUHOW, metabolically unhealthy overweight; MUHO, metabolically unhealthy obesity.

^a^ Kruskal–Wallis rank sum test; Pearson's Chi‐squared test.

**FIGURE 2:**
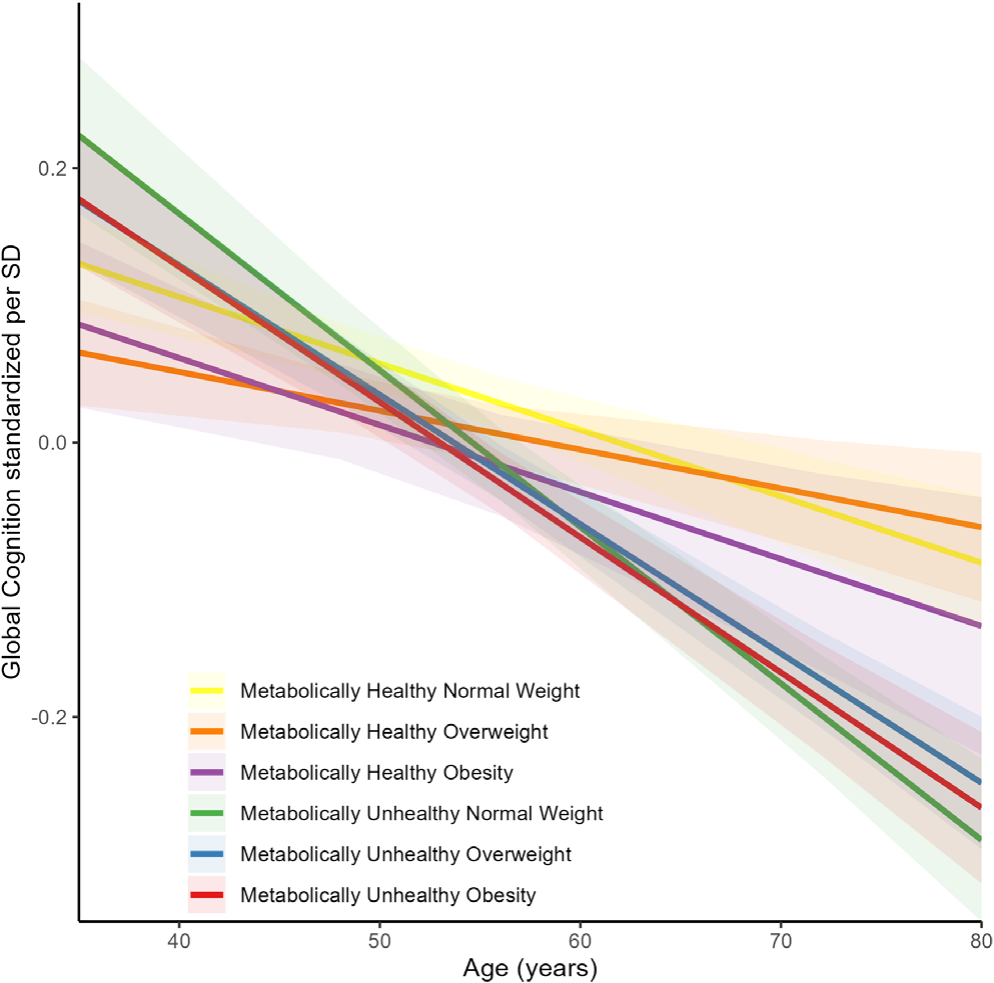
Association between metabolic phenotypes and global cognitive decline during 8 years of follow‐up. Linear mixed model adjusted for age, sex, race, marital status, education level, depressive symptoms, alcohol use, smoker status, and physical inactivity at baseline. Inverse probability of attrition weighting (IPAW) was used to adjust for attrition related to mortality and missing participation at each wave. The shaded area represents the 95% confidence interval. SD, standard deviation.

Compared to MHNW, metabolically unhealthy categories were associated with faster global cognitive decline in the final model (MUHNW: β = −0.053; 95% CI: −0.074, −0.031; *p* < 0.001; MUHOW: β = −0.037; 95% CI: −0.055, −0.019; *p* < 0.001; MUHO: β = −0.040; 95% CI: −0.060, −0.021; *p* < 0.001). In contrast, MHO or MHOW showed no association with global cognitive decline (MHOW: β = 0.016; 95% CI: −0.002, 0.034; *p* = 0.081; MHO: β = 0.000; 95% CI: −0.027, 0.026; *p* = 0.981) (Table 2 and Figure 2). In these models, negative β coefficients indicate faster decline in global cognition over the follow‐up. These findings remained directionally consistent when metabolic health was defined using NCEP‐ATP III criteria and in sensitivity analysis with imputed cognitive scores (Tables S3 and S4). No mediation by CRP levels in these associations was identified (Table S5). Variance components from the main model are provided in Table S6.

**TABLE 2:** Associations between metabolic phenotypes and cognitive decline over 8 years of follow‐up (*n* = 12 795).

	Unadjusted			Model 1			Model 2		
	β	95% CI	*p*‐value	β	95% CI	*p*‐value	β	95% CI	*p*‐value
Global cognition									
MHNW * age	[Ref.]	[Ref.]	[Ref.]	[Ref.]	[Ref.]	[Ref.]	[Ref.]	[Ref.]	[Ref.]
MHOW * age	0.028	0.009, 0.048	**0.004**	0.017	−0.001, 0.035	0.067	0.016	−0.002, 0.034	0.081
MHO * age	0.008	−0.020, 0.037	0.561	0.000	−0.026, 0.027	0.998	0.000	−0.027, 0.026	0.981
MUHNW * age	−0.053	−0.075, −0.030	**<0.001**	−0.053	−0.074, −0.032	**<0.001**	−0.053	−0.074, −0.031	**<0.001**
MUHOW * age	−0.026	−0.045, −0.007	**0.009**	−0.036	−0.054, −0.018	**<0.001**	−0.037	−0.055, −0.019	**<0.001**
MUHO * age	−0.034	−0.055, −0.013	**0.001**	−0.041	−0.060, −0.021	**<0.001**	−0.040	−0.060, −0.021	**<0.001**
Memory									
MHNW * age	[Ref.]	[Ref.]	[Ref.]	[Ref.]	[Ref.]	[Ref.]	[Ref.]	[Ref.]	[Ref.]
MHOW * age	0.038	0.011, 0.065	**0.005**	0.024	−0.002, 0.050	0.072	0.023	−0.003, 0.049	0.089
MHO * age	0.024	−0.015, 0.063	0.225	0.013	−0.026, 0.051	0.517	0.012	−0.026, 0.051	0.520
MUHNW * age	−0.068	−0.099, −0.037	**<0.001**	−0.070	−0.101, −0.040	**<0.001**	−0.072	−0.102, −0.041	**<0.001**
MUHOW * age	−0.017	−0.044, 0.010	0.218	−0.030	−0.057, −0.004	**0.023**	−0.033	−0.059, −0.007	**0.014**
MUHO * age	−0.022	−0.051, 0.007	0.135	−0.033	−0.061, −0.005	**0.021**	−0.034	−0.063, −0.006	**0.017**
TMT‐B									
MHNW * age	[Ref.]	[Ref.]	[Ref.]	[Ref.]	[Ref.]	[Ref.]	[Ref.]	[Ref.]	[Ref.]
MHOW * age	0.010	−0.022, 0.042	0.541	0.002	−0.027, 0.031	0.885	0.002	−0.027, 0.031	0.906
MHO * age	−0.012	−0.058, 0.034	0.603	−0.010	−0.053, 0.032	0.639	−0.010	−0.052, 0.032	0.645
MUHNW * age	−0.033	−0.070, 0.005	0.086	−0.031	−0.065, 0.003	0.077	−0.027	−0.061, 0.007	0.122
MUHOW * age	−0.041	−0.073, −0.009	**0.011**	−0.055	−0.085, −0.026	**<0.001**	−0.053	−0.083, −0.024	**<0.001**
MUHO * age	−0.062	−0.096, −0.028	**<0.001**	−0.067	−0.099, −0.035	**<0.001**	−0.064	−0.096, −0.032	**<0.001**
Verbal fluency									
MHNW * age	[Ref.]	[Ref.]	[Ref.]	[Ref.]	[Ref.]	[Ref.]	[Ref.]	[Ref.]	[Ref.]
MHOW * age	0.018	−0.008, 0.045	0.180	0.007	−0.018, 0.032	0.596	0.006	−0.019, 0.032	0.626
MHO * age	−0.021	−0.060, 0.019	0.303	−0.025	−0.062, 0.012	0.185	−0.026	−0.063, 0.011	0.172
MUHNW * age	−0.031	−0.062, 0.000	0.050	−0.035	−0.064, −0.006	**0.019**	−0.033	−0.062, −0.004	**0.026**
MUHOW * age	−0.024	−0.051, 0.003	0.080	−0.036	−0.061, −0.011	**0.005**	−0.035	−0.060, −0.010	**0.006**
MUHO * age	−0.030	−0.059, −0.001	**0.042**	−0.038	−0.065, −0.011	**0.006**	−0.036	−0.063, −0.009	**0.009**

*Note*: Model 1: linear mixed‐model with random slopes and intercepts adjusted for age, sex, race, marital status, and education level at baseline. Model 2: linear mixed‐model with random slopes and intercepts adjusted for age, sex, race, marital status, education level, alcohol use, smoker status, physical inactivity, and depressive symptoms at baseline. Inverse probability weighting was used to adjust all models for attrition related to mortality and missing participation at each wave. *p* values < 0.05 are represented in bold.

Abbreviations: CI, confidence interval; MHNW, metabolically healthy normal weight; MHOW, metabolically healthy overweight; MHO, metabolically healthy obesity; MUHNW, metabolically unhealthy normal weight; MUHOW, metabolically unhealthy overweight; MUHO, metabolically unhealthy obesity; TMT‐B, trail‐making test part B.

When MHO was used as the reference category, MUHNW, MUHOW, and MUHO were also associated with faster global cognitive decline (MUHNW: β = −0.052; 95% CI: −0.081, −0.023; *p* < 0.001; MUHOW: β = −0.036; 95% CI: −0.064, −0.009; *p* = 0.009; MUHO: β = −0.040; 95% CI: −0.068, −0.012; *p* = 0.006), whereas MHNW and MHOW were not (MHNW: β = 0.000; 95% CI: −0.026, 0.027; *p* = 0.981; MHOW: β = 0.016; 95% CI: −0.011, 0.044; *p* = 0.236) (Table S7).

When MUHO was used as the reference category, MHNW, MHOW and MHO were associated with a slower rate of global cognitive decline (MHNW: β = 0.040; 95% CI: 0.021, 0.060; *p* < 0.001; MHOW: β = 0.056; 95% CI: 0.036, 0.077; *p* < 0.001; MHO: β = 0.040; 95% CI: 0.012, 0.068; p = 0.006). In contrast, no differences in cognitive decline were observed between MUHO and the other metabolically unhealthy phenotypes (MUHNW: β = −0.012; 95% CI: −0.036, 0.011; *p* = 0.292; MUHOW: β = 0.003; 95% CI: −0.017, 0.024; *p* = 0.746) (Table S8).

No association between BMI and cognitive decline was identified (β = −0.001; 95% CI: −0.002, 0.000; *p* = 0.170) (Table S9). However, higher WHR was associated with faster cognitive decline (β = −0.020, 95% CI: −0.026, −0.014, *p* < 0.001) (Table S10).

The association between metabolic phenotypes and cognitive decline was modified by race (*p* < 0.001) and sex (*p* = 0.004), but not by older age status (*p* = 0.059). In analyses stratified by race, MUHNW, MUHOW, and MUHO were associated with faster cognitive decline than MHNW in White participants (MUHNW: β = −0.052; 95% CI: −0.079, −0.024; p < 0.001; MUHOW: β = −0.054; 95% CI: −0.077, −0.030; *p* < 0.001; MUHO: β = −0.034; 95% CI: −0.060, −0.008; *p* = 0.009), but only MUHNW was associated with faster cognitive decline in Black/Mixed race participants (MUHNW: β = −0.038; 95% CI: −0.073, −0.003; *p* = 0.035; MUHOW: β = −0.007; 95% CI: −0.038, 0.023; *p* = 0.643; MUHO: β = −0.028; 95% CI: −0.060, 0.004; *p* = 0.082). MHO and MHOW were not associated with cognitive decline in either group (Table S11).

In analyses stratified by sex, MUHO and MUHNW were associated with faster cognitive decline in both men (MUHNW: β = −0.039; 95% CI: −0.072, −0.007; *p* = 0.018; MUHO: β = −0.034; 95% CI: −0.066, −0.002; p = 0.035) and women (MUHNW: β = −0.054; 95% CI: −0.082, −0.026; *p* < 0.001; MUHO: β = −0.040; 95% CI: −0.065, −0.015; *p* = 0.002) (Table S12). MUHOW was associated with a faster cognitive decline only in women (Women: β = −0.044; 95% CI: −0.069, −0.019; p < 0.001; Men: β = −0.018; 95% CI: −0.045, 0.010; *p* = 0.215). MHOW was associated with slower cognitive decline in men and was not associated with cognitive decline in women (Men: β = 0.042; 95% CI: 0.012, 0.072; *p* = 0.006; Women: β = 0.001; 95% CI: −0.022, 0.023; *p* = 0.945). MHO was not associated with cognitive decline in either group (Men: β = 0.031; 95% CI: −0.019, 0.080; *p* = 0.224; Women: β −0.015; 95% CI: −0.046, 0.015; *p* = 0.328).

## 4 DISCUSSION

In this large cohort study, metabolically unhealthy normal weight, overweight, and obesity were associated with accelerated global cognitive decline over an eight‐year follow‐up, while metabolically healthy phenotypes had no association. No evidence of mediation by CRP levels was identified. Similarly, BMI was not associated with cognitive decline, whereas a higher WHR was. Sex and race differences were observed.

Our findings suggest that metabolic dysfunction may be a stronger predictor of cognitive decline than excess adiposity. The observed association between WHR, but not BMI, and cognitive decline further supports this interpretation, as WHR is more closely associated with abdominal obesity, a known risk factor for metabolic syndrome.^40^ Taken together, these findings argue against a model in which excess body fat directly promotes neurodegeneration and instead support an interpretation in which obesity is associated with cognitive decline primarily through metabolic dysfunction, potentially involving neurovascular injury. In line with this hypothesis, prior studies have suggested that hypertension and diabetes mediate the relationship between obesity and cognitive impairment, while neuroimaging studies have linked visceral adiposity and higher body fat percentage to vascular brain injury and poorer cognitive performance.^41^, ^42^, ^43^, ^44^ Consistent with this interpretation, optimal cardiovascular health, particularly blood glucose and blood pressure regulation, has been associated with slower cognitive decline.^45^

Evidence from other large population‐based cohorts aligns with our findings. Prior studies have reported that WHR, but not BMI, was associated with poorer cognitive performance and an increased risk of dementia.^2^, ^3^, ^46^ Similarly, analyses from the Swedish Twin Registry found no evidence of mediation by CRP in the association between adiposity and dementia.^46^ Although CRP is a widely used marker of low‐grade systemic inflammation, it captures only one dimension of the complex inflammatory and vascular processes potentially linking metabolic dysfunction to cognitive decline.^47^ Other cytokines, such as interleukin‐6 (IL‐6) and tumour necrosis factor‐α (TNF‐α), have been shown to be stronger predictors of cognitive decline in midlife than CRP.^48^ In addition, experimental and epidemiological evidence supports the plausibility of neurovascular mechanisms, such as endothelial dysfunction, impaired neurovascular coupling, and increased blood–brain barrier permeability, that may mediate the association between metabolic dysfunction and cognitive decline but were not assessed in this study.^49^, ^50^, ^51^ These assumed mediation pathways are represented in Figure S1. Such pathways may operate independently of CRP, or CRP may not be sufficiently sensitive to capture the underlying inflammatory and vascular processes, which could partly explain the absence of detectable mediation. Therefore, reliance on CRP as a single inflammatory marker represents an important limitation.

Unlike what has been reported for cardiovascular mortality, applying a more stringent definition of metabolic health did not change the overall interpretation of the association between MHO and cognitive decline in our study.^12^ Agreement between the traditional NCEP‐ATP III and the newer, more stringent definition was substantial, although a meaningful proportion of participants were reclassified. While effect estimates varied in magnitude across definitions, confidence intervals overlapped and the direction of associations remained consistent. Differences in estimates may also reflect the smaller analytic sample available for the NCEP‐ATP III definition due to additional missingness, which can reduce precision and introduce modest selection differences compared with the main classification.

The findings from our study differ from previous research that reported an increased risk of dementia among individuals with MHO compared to those with metabolically healthy non‐obesity in the Whitehall II cohort.^14^ A potential explanation for this discrepancy may be the follow‐up duration, as participants in that study were followed for 27 years, whereas our study had an eight‐year follow‐up. Supporting this explanation, reports from the Framingham Offspring Study (13 years of follow‐up) also found no change in cognitive function over time between MHO and MHNW individuals.^13^ This raises the possibility that MHO represents a transient state with an elevated risk of progressing to metabolically unhealthy phenotypes over time, a pattern that has been observed in other populations.^52^, ^53^, ^54^ Such phenotype transitions could attenuate observed associations over shorter follow‐up periods, particularly when metabolic status is assessed only at baseline. Consistent with this framework, prior analyses from ELSA‐Brasil that captured a broader exposure window showed that weight gain trajectories from early to midlife were associated with accelerated cognitive decline.^55^ An additional explanation for the discrepancies across this study and others may involve selection bias related to differential exclusion of participants at baseline.

The observed racial differences, where MUHNW but not MUHOW or MUHO were associated with accelerated cognitive decline among Black/Mixed race participants, could reflect underlying socio‐environmental and early‐life influences, including cumulative socioeconomic disadvantage, early‐life adversity, and differential access to healthcare, which were not fully captured in our study. Selection mechanisms related to differential survival and study retention across race groups may also have contributed to these patterns. In particular, Black/Mixed individuals with more severe cardiometabolic burden, such as those classified as MUHO or MUHOW, may have experienced higher competing mortality or earlier clinical deterioration, reducing their likelihood of surviving long enough to be enrolled or retained in the cohort and thereby selectively depleting the highest‐risk strata, which would tend to attenuate the observed associations toward the null. Although IPAW was applied to mitigate bias related to loss to follow‐up, it cannot fully address selection processes operating before baseline enrolment or those driven by unmeasured determinants of survival and retention. These differences may also partly reflect limited statistical power within specific phenotype–race strata due to reduced sample size. Another possible explanation involves racial differences in fat distribution, as Black individuals generally have lower visceral and greater peripheral adiposity, potentially reducing metabolic risks.^56^, ^57^ Regarding sex differences, the observed associations were largely consistent across men and women, except in the overweight categories, where differences may be attributed to misclassification of weight status due to sex‐based variations in fat and muscle mass distribution.

This study has several strengths. The eight‐year follow‐up allowed for evaluation of cognitive decline rather than relying on cross‐sectional data. Studying a mostly middle‐aged cohort reduced the risk of reverse causation, a greater concern in older adults where weight loss may result from neurodegeneration or other chronic diseases related to dementia risk.^25^, ^58^ The use of both traditional and stringent metabolic health definitions and the inclusion of BMI and WHR strengthened the evaluation of metabolic obesity phenotypes. The diverse cohort provided insights into sex and racial differences. However, some limitations should be acknowledged. The follow‐up period may be insufficient to fully capture long‐term cognitive trajectory risk, and metabolic phenotype transitions over time were not accounted for. The lack of neuroimaging data limits insights into cerebrovascular mechanisms, and residual confounding from early‐life factors or genetic predispositions may have influenced findings. Although mitigated by midlife sampling, potential reverse causation cannot be fully excluded, as early subclinical cognitive changes may still influence lifestyle behaviours or metabolic factors. Furthermore, the cohort consists of relatively well‐educated public servants, which may limit generalizability. Finally, hearing impairment and dietary patterns were not explicitly adjusted for and may represent an additional source of confounding. Multiple testing correction was not applied to the stratified analyses (sex, race), and these results should therefore be interpreted with caution.

In conclusion, our findings suggest that metabolic dysfunction may be a stronger predictor of cognitive decline than excess body weight per se. Dementia prevention strategies may benefit from prioritizing early identification and management of metabolic dysfunction across all weight categories, and from considering WHR as a complementary measure to BMI in cognitive risk stratification. Future research should consider metabolic state transitions over time, extended follow‐up periods, neuroimaging, adipokines, and genetic data to further elucidate the underlying mechanisms and racial differences in these associations.

## AUTHOR CONTRIBUTIONS

Paulo Henrique Lazzaris Coelho and Claudia Kimie Suemoto conceptualized the study. Paulo Henrique Lazzaris Coelho analysed data and drafted the manuscript. Natalia Gomes Gonçalves supervised the statistical analysis, and Claudia Kimie Suemoto oversaw the project. Paulo Andrade Lotufo and Isabela Martins Bensenor provided administrative support, funding, and designed the data acquisition. All authors reviewed and approved the manuscript.

## FUNDING INFORMATION

ELSA‐Brasil study was supported by the Brazilian Ministry of Health, the Ministry of Science, Technology and Innovation, and the National Council for Scientific and Technological Development (CNPq). Natalia Gomes Gonçalves receives AARF‐D fellowship from the Alzheimer's Association (grant 23AARFD‐1029257). Claudia Kimie Suemoto receives Alzheimer's Association (grant AARG‐20‐678884 and 24CBIDR‐1185483) and CNPq productivity fellowship (303883/2021‐9). Paulo Caramelli receives CNPq productivity fellowship (313678/2021‐9).

## CONFLICT OF INTEREST STATEMENT

The authors declare no competing financial interests.

## Supporting information


**DATA S1:** Supporting information.

## Data Availability

Data that support the findings of this study were used under licence and are not publicly available. However, they are available from the authors upon reasonable request and with permission of Claudia Kimie Suemoto.
