# Supplementary material for: Association of metabolic obesity phenotypes with cognitive decline in the ELSA‐Brasil study
Source: Diabetes Obes Metab. 2026 Jan 20;28(4):2849–58. doi: 10.1111/dom.70466 (PMC12992203; doi:10.1111/dom.70466)
Supplement: Supplementary file 1 — DATA S1: Supporting information. [file DOM-28-2849-s001.docx]

**Association of metabolic obesity phenotypes with cognitive decline in the ELSA-Brasil study – Supplementary Material**

[SUPPLEMENTARY TEXT 3](#_Toc216622641)

[Metabolic health defined by NCEP ATP III 3](#_Toc216622642)

[Anthropometric measurements 3](#_Toc216622643)

[Diabetes definition 3](#_Toc216622644)

[Cognitive evaluation 3](#_Toc216622645)

[Linear mixed models 4](#_Toc216622646)

[Inverse probability of attrition weighting 5](#_Toc216622647)

[Mediation analyses 5](#_Toc216622648)

[Sensitivity analyses 6](#_Toc216622649)

[SUPPLEMENTARY FIGURES 7](#_Toc216622650)

[SUPPLEMENTARY TABLES 8](#_Toc216622651)

[Supplementary Table 1. Overlap between strict and NCEP-ATP III metabolic obesity phenotypes (n = 12,795) 8](#_Toc216622652)

[Cohen’s unweighted κ = 0.64 (95% CI: 0.63 - 0.65) 8](#_Toc216622653)

[Strict metabolic health definition based on Schulze & Stefan (2024). 8](#_Toc216622654)

[Supplementary Table 2. Comparison of baseline sociodemographic, clinical characteristics, and cognitive assessment between included and excluded participants (n = 15,105). 8](#_Toc216622655)

[Supplementary Table 3. Associations between metabolic phenotypes defined by NCEP-ATP III criteria and cognitive decline during eight years of follow-up (n = 12,795). 10](#_Toc216622656)

[Supplementary Table 4. Associations between metabolic phenotypes and cognitive decline during eight years of follow-up using the last observation carried backward from wave 3 for missing cognitive performance information of participants < 55 years in wave 2 (n = 12,795). 11](#_Toc216622657)

[Supplementary Table 5. Mediation by CRP levels of the association between metabolic phenotypes and cognitive decline (n = 12,795). 12](#_Toc216622658)

[Supplementary Table 6. Random-effects variance components from the main linear mixed model 12](#_Toc216622659)

[Supplementary Table 7. Associations between metabolic phenotypes and global cognitive decline during eight years of follow-up, using MHO as the reference (n = 12,795). 13](#_Toc216622660)

[Supplementary Table 8. Associations between metabolic phenotypes and global cognitive decline during eight years of follow-up, using MUHO as the reference (n = 12,795). 14](#_Toc216622661)

[Supplementary Table 9. Association between body mass index and global cognitive decline during eight years of follow-up (n = 12,795). 14](#_Toc216622662)

[Supplementary Table 10. Association between WHR and global cognitive decline during eight years of follow-up (n = 12,795). 15](#_Toc216622663)

[Supplementary Table 11. Associations between metabolic phenotypes and cognitive decline during eight years of follow-up stratified by race (n = 12,355). 16](#_Toc216622664)

[Supplementary Table 12. Associations between metabolic phenotypes and cognitive decline during eight years of follow-up stratified by sex (n = 12,795). 16](#_Toc216622665)

# SUPPLEMENTARY TEXT

Metabolic health defined by NCEP ATP III

According to this definition, metabolic health was determined by the presence of fewer than two metabolic risk factors: (1) triglycerides ≥150 mg/dL or lipid-lowering medication use, (2) HDL cholesterol <50 mg/dL for women or <40 mg/dL for men, (3) systolic blood pressure ≥130 mmHg, diastolic blood pressure ≥85 mmHg, or antihypertensive medication use, and (4) diabetes. (1) Participants were classified into the same six phenotype categories (MHO, MUHO, MHOW, MUHOW, MHNW, and MUHNW) under this traditional definition.

Anthropometric measurements

Weight (in kg) was measured using a Toledo weight scale and height (in cm) with a SECA-SE-216 stadiometer. Waist circumference (WC, in cm) was measured at the midpoint between the lower edge of the rib cage and the iliac crest along the mid-axillary line. Hip circumference was measured at the level of the maximum protrusion of the gluteal muscles. Both measures were taken using a Mabis Gulick non-stretchable tape measure and the average of two measurements in each circumference was used for analysis. From these measurements, the body mass index (BMI, kg/m²) was calculated by dividing the weight by the square of the height in meters; and the waist-to-hip ratio (WHR) by dividing the WC by the hip circumference. (2) During the measurements, participants wore only a standardized study gown over their underwear and did not wear shoes.

Diabetes definition

Diabetes was defined by fasting glucose ≥ 7.0 mmol/L, 2h-postprandial 75g glucose ≥ 11.1 mmol/L, glycated hemoglobin ≥ 6.5%, use of hypoglycemic medication or insulin, or self-reported medical diagnosis.

Cognitive evaluation

The CERAD word list test evaluates immediate recall, delayed recall, and recognition of a 10-word list.(3) For the immediate recall test, cards with 10 unrelated words were presented to participants. Immediately after reading the 10 words, they were asked to recall as many words as possible. The test was repeated three times and the immediate recall score was calculated as the sum of recalled words from the three trials (0 – 30 words). After approximately five minutes, during which participants performed another cognitive test, they were instructed to recall the maximum number of words from the list. The delayed recall score was calculated as the number of words recalled (0 – 10 words). After the delayed recall test, participants were asked to recognize the previously presented words from a list of 20 words that contained the 10 original words and 10 different words. The recognition score was calculated as the number of correctly recalled words minus the number of intrusions (0 – 10 words).

For the phonemic verbal fluency test, participants were requested to verbally list, in one minute, as many words as possible starting with the letter F (letter A was used in wave 2).(4) For the semantic verbal fluency test, participants were requested to verbally list as many animals as possible in one minute (vegetable category was used in wave 2).(4) The score for each test was the number of correct words produced. Verbal fluency test results were harmonized across waves to account for the different categories and letters used.(5)

In the trail-making test, participants were instructed to connect numbers and letters in ascending and alternating order (e.g., 1-A-2-B-3-C). (6) Participants had a learning trial first and, after understanding the task, they received a page with numbers from 1 to 13 and letters from A to L. The score of this task was the time in seconds that they took to complete the task. If the participant committed an error, the interviewer instructed them to return to the last correct point without stopping the chronometer.

For the first wave, each cognitive test score was transformed into a Z-score by subtracting the mean and dividing by the standard deviation (SD). For the second and third waves, test scores were standardized relative to the first wave means and standard deviations. The memory Z-score was computed by averaging the Z-scores for the immediate recall, delayed recall, and recognition tests. Similarly, the verbal fluency Z-score was obtained by averaging the Z-scores for the semantic and phonemic tests. The trail-making Z-score was multiplied by ‑1 because higher scores in this test denote poorer performance, while higher scores in the other tests indicate better performance. A global cognition Z-score was computed by averaging the Z-scores of the six tests.

Linear mixed models

To investigate the association between metabolic phenotypes and cognitive decline, linear mixed models (LMM) with random intercepts and slopes were used, with age at each wave as the timescale. The primary analyses used MHNW as the reference category. To further assess the role of metabolic dysfunction within obesity, we conducted a supplementary analysis using MHO as the reference category. This allowed for a direct comparison between metabolically healthy and unhealthy obesity. The timescale was rescaled to correspond to the median follow-up period (eight years), thereby making a one-unit change in the timescale correspond to eight years. This scaling facilitated the interpretation of coefficients, allowing them to reflect cognitive changes over eight years. The first model was adjusted for baseline age, sex, race, marital status, and education level. The second and final model was further adjusted for baseline alcohol consumption, smoking status, physical inactivity, and depressive symptoms. The same modeling approach was applied using BMI and WHR as continuous exposures instead of metabolic phenotypes. For these exposures, a third model adjusted for hypertension, diabetes, dyslipidemia, and cardiovascular disease, although it must be interpreted cautiously as these variables are potential mediators in the association of BMI and WHR with cognitive decline. These covariates were not included in metabolic phenotype models to avoid overadjustment, as they are part of the definition of metabolic health. WHR was standardized by scaling to its standard deviation to improve numerical stability, hence the effect estimates correspond to a one-SD increase in WHR.

Inverse probability of attrition weighting

Inverse Probability of Attrition Weighting (IPAW) was applied to address potential attrition bias. (7) Weights were calculated separately for survival and participation across waves using logistic regression models that estimated the probability of being alive and participating in each wave. Each model included baseline exposure (metabolic phenotype, BMI, or WHR), baseline covariates (age, sex, race, marital status, education), and wave-specific covariates from the previous wave (global cognition, smoking, alcohol use, physical inactivity, depressive symptoms, hypertension, diabetes, dyslipidemia, and cardiovascular disease). Model fit was assessed using the Hosmer-Lemeshow test (p > 0.05 for all models). Weights were stabilized using baseline covariates and truncated at the 1st and 99th percentiles to minimize the influence of extreme values. Final weights were derived by multiplying survival and participation weights across waves and were incorporated into the linear mixed models.

Mediation analyses

Mediation of the association between metabolic phenotypes and cognitive decline by baseline CRP levels was evaluated using the difference-of-coefficients method. (8) Two LMMs were fitted: the total effect model (i.e., the final model described above) and the direct effect model, which additionally included baseline CRP levels and its interaction with the timescale. The coefficients for the exposure–timescale interaction in these models represent the total and direct effects, respectively, and the indirect effect was computed as their difference. The proportion mediated was calculated as the indirect effect divided by the total effect. Confidence intervals were obtained via 1,000 bootstrap iterations. This mediation analysis assumes that: (1) exposure, mediator, and outcome occur in a temporal sequence; (2) there are no unmeasured mediator-outcome confounders; and (3) there is no mediator-exposure interaction.

Sensitivity analyses

Sensitivity analyses were performed to address the potential bias from excluding participants under 55 years old at the second wave and are described in the supplementary material. We applied the last observation carried backward method, imputing cognitive Z-scores using data from the third wave. This approach was considered conservative, since cognitive function generally declines over time, and thus, carrying scores backward minimizes the risk of overestimating cognitive performance in the imputed data.

1. Machado-Fragua MD, Sabia S, Fayosse A, Hassen CB, van der Heide F, Kivimaki M, et al. Is metabolic-healthy obesity associated with risk of dementia? An age-stratified analysis of the Whitehall II cohort study. BMC Med. 2023 Nov 14;21:436.

2. Benseñor, Isabela Judith Martins, Schmidt MI, Griep RH. Manuais do ELSA-Brasil: Antropometria [Internet]. Centro de Investigação ELSA-Brasil; 2016. Available from: http://elsabrasil.org/pesquisadores/manuais-de-procedimentos/

3. Moms JC, Heyman A, Mohs RC, Hughes JP, van Belle G, Fillenbaum G, et al. The Consortium to Establish a Registry for Alzheimer’s Disease (CERAD). Part I. Clinical and neuropsychological assesment of Alzheimer’s disease. Neurology. 1989 Sept;39(9):1159–1159.

4. Strauss E, Sherman EMS, Spreen O. A compendium of neuropsychological tests: Administration, norms, and commentary, 3rd ed. New York, NY, US: Oxford University Press; 2006. xvii, 1216 p. (A compendium of neuropsychological tests: Administration, norms, and commentary, 3rd ed).

5. Bertola L, Benseñor IM, Gross AL, Caramelli P, Barreto SM, Moreno AB, et al. Longitudinal measurement invariance of neuropsychological tests in a diverse sample from the ELSA-Brasil study. Braz J Psychiatry. 2020 Sept 28;43(3):254–61.

6. Greenlief CL, Margolis RB, Erker GJ. Application of the Trail Making Test in Differentiating Neuropsychological Impairment of Elderly Persons. Percept Mot Skills. 1985 Dec 1;61(3_suppl):1283–9.

7. Weuve J, Tchetgen Tchetgen EJ, Glymour MM, Beck TL, Aggarwal NT, Wilson RS, et al. Accounting for bias due to selective attrition: The example of smoking and cognitive decline. Epidemiology. 2012 Jan;23(1):119–28.

8. VanderWeele TJ. Mediation Analysis: A Practitioner’s Guide. Annu Rev Public Health. 2016 Mar 18;37(1):17–32.

# SUPPLEMENTARY FIGURES

**Supplementary Figure 1. Directed acyclic graph illustrating assumed pathways between metabolic dysfunction and cognitive decline.**

Metabolic dysfunction

Cognitive decline

CRP

Baseline confounders

Alternative inflammatory pathways

Neurovascular mechanisms

Solid arrows represent pathways evaluated in this study, including mediation through C-reactive protein (CRP). Dashed arrows indicate potential alternative inflammatory pathways (e.g., IL-6 and TNF-α) and neurovascular pathways (including endothelial dysfunction, impaired neurovascular coupling, and increased blood–brain barrier permeability) that were not assessed in this study. Baseline confounders represent sociodemographic, lifestyle, and clinical factors.

# SUPPLEMENTARY TABLES

Supplementary Table 1. Overlap between strict and NCEP-ATP III metabolic obesity phenotypes (n = 12,795)

|  | NCEP: MHNW | NCEP: MHO | NCEP: MHOW | NCEP: MHUW | NCEP: MUHNW | NCEP: MUHO | NCEP: MUHOW | NCEP: MUHUW |  |
| --- | --- | --- | --- | --- | --- | --- | --- | --- | --- |
|  |  |  |  |  |  |  |  |  |  |
| Strict: MHNW | 2,974 | 0 | 0 | 0 | 254 | 0 | 0 | 0 |  |
| Strict: MHO | 0 | 696 | 0 | 0 | 0 | 157 | 0 | 0 |  |
| Strict: MHOW | 0 | 0 | 2,189 | 0 | 0 | 0 | 377 | 0 |  |
| Strict: MHUW | 0 | 0 | 0 | 0 | 0 | 0 | 0 | 0 |  |
| Strict: MUHNW | 941 | 0 | 0 | 0 | 577 | 0 | 0 | 0 |  |
| Strict: MUHO | 0 | 826 | 0 | 0 | 0 | 1,216 | 0 | 0 |  |
| Strict: MUHOW | 0 | 0 | 1,136 | 0 | 0 | 0 | 1,452 | 0 |  |
| Strict: MUHUW | 0 | 0 | 0 | 0 | 0 | 0 | 0 | 0 |  |

Cohen’s unweighted κ = 0.64 (95% CI: 0.63 - 0.65)

Strict metabolic health definition based on Schulze & Stefan (2024).

Supplementary Table 2. Comparison of baseline sociodemographic, clinical characteristics, and cognitive assessment between included and excluded participants (n = 15,105).

|  | **Included**  **(n =** 12,795*)* | **Excluded**  **(n** = 2,310) | **p-value²** |
| --- | --- | --- | --- |
| Age (years) | 51.5 (8.9) | 55.4 (9.3) | <0.001 |
| Women | 55.1% | 50.3% | <0.001 |
| Race |  |  | <0.001 |
| White | 52.9% | 48.2% |  |
| Black/Brown | 43.7% | 47.6% |  |
| Other | 3.4% | 4.3% |  |
| College or more | 55.4% | 37.3% | <0.001 |
| Married | 66.4% | 64.7% | 0.12 |
| Smoking status |  |  | <0.001 |
| Never | 57.8% | 52.0% |  |
| Former | 29.6% | 32.2% |  |
| Current | 12.6% | 15.8% |  |
| Alcohol use |  |  | <0.001 |
| Never | 10.1% | 14.1% |  |
| Former | 19.0% | 26.4% |  |
| Current | 70.9% | 59.4% |  |
| Depressive symptoms | 12.7% | 15.7% | <0.001 |
| Physical inactivity | 74.9% | 80.2% | <0.001 |
| C-Reactive Protein (mg/L) | 2.8 (4.5) | 3.0 (5.0) | 0.13 |
| Metabolic phenotype |  |  | <0.001 |
| Metabolically healthy underweight | 0.0% | 4.7% |  |
| Metabolically unhealthy underweight | 0.0% | 1.5% |  |
| Metabolically healthy normal weight | 25.2% | 15.3% |  |
| Metabolically unhealthy normal weight | 11.9% | 14.1% |  |
| Metabolically healthy overweight | 20.1% | 13.4% |  |
| Metabolically unhealthy overweight | 20.2% | 26.6% |  |
| Metabolically healthy obesity | 6.7% | 4.0% |  |
| Metabolically unhealthy obesity | 16.0% | 20.4% |  |
| Semantic verbal fluency | 18.8 (5.2) | 16.2 (5.4) | <0.001 |
| Phonemic verbal fluency | 12.8 (4.4) | 10.4 (4.9) | <0.001 |
| Immediate memory | 21.3 (3.8) | 19.9 (4.4) | <0.001 |
| Word recognition | 9.6 (0.9) | 9.4 (1.2) | <0.001 |
| Late recall | 7.0 (2.0) | 6.4 (2.2) | <0.001 |
| Trail Making Test version B (seconds) | 122.4 (85.4) | 127.8 (94.3) | 0.3 |

Data are presented as mean (SD) for continuous variables and percentages for categorical variables. BMI = Body Mass Index

¹ Wilcoxon rank sum test; Pearson’s Chi-squared test

Supplementary Table 3. Associations between metabolic phenotypes defined by NCEP-ATP III criteria and cognitive decline during eight years of follow-up (n = 12,795).

|  | **Unadjusted** | | | **Model 1** | | | **Model 2** | | |
| --- | --- | --- | --- | --- | --- | --- | --- | --- | --- |
|  | **Beta** | **95% CI** | **p-value** | **Beta** | **95% CI** | **p-value** | **Beta** | **95% CI** | **p-value** |
| **Global Cognition** |  |  |  |  |  |  |  |  |  |
| mhnw * age | [Ref.] | [Ref.] | [Ref.] | [Ref.] | [Ref.] | [Ref.] | [Ref.] | [Ref.] | [Ref.] |
| mhow * age | 0.018 | 0.001, 0.035 | 0.042 | 0.011 | -0.005, 0.027 | 0.171 | 0.010 | -0.006, 0.026 | 0.230 |
| mho * age | -0.002 | -0.024, 0.020 | 0.831 | -0.008 | -0.028, 0.013 | 0.460 | -0.008 | -0.029, 0.012 | 0.424 |
| muhnw * age | -0.062 | -0.089, -0.034 | <0.001 | -0.067 | -0.093, -0.041 | **<0.001** | -0.067 | -0.093, -0.041 | **<0.001** |
| muhow * age | -0.007 | -0.028, 0.014 | 0.516 | -0.022 | -0.041, -0.003 | **0.026** | -0.023 | -0.042, -0.004 | **0.020** |
| muho * age | -0.022 | -0.045, 0.001 | 0.062 | -0.032 | -0.053, -0.010 | **0.004** | -0.031 | -0.053, -0.010 | **0.004** |
| **Memory** |  |  |  |  |  |  |  |  |  |
| mhnw * age | [Ref.] | [Ref.] | [Ref.] | [Ref.] | [Ref.] | [Ref.] | [Ref.] | [Ref.] | [Ref.] |
| mhow * age | 0.033 | 0.009, 0.057 | 0.006 | 0.024 | 0.001, 0.047 | 0.038 | 0.022 | -0.001, 0.045 | 0.057 |
| mho * age | 0.017 | -0.014, 0.048 | 0.277 | 0.007 | -0.022, 0.037 | 0.628 | 0.006 | -0.023, 0.036 | 0.674 |
| muhnw * age | -0.077 | -0.116, -0.039 | <0.001 | -0.089 | -0.126, -0.052 | <0.001 | -0.090 | -0.127, -0.053 | **<0.001** |
| muhow * age | 0.002 | -0.026, 0.031 | 0.865 | -0.018 | -0.046, 0.009 | 0.194 | -0.021 | -0.048, 0.007 | 0.141 |
| muho * age | -0.013 | -0.045, 0.019 | 0.424 | -0.028 | -0.059, 0.002 | 0.071 | -0.030 | -0.060, 0.001 | 0.059 |
| **TMT-B** |  |  |  |  |  |  |  |  |  |
| mhnw * age | [Ref.] | [Ref.] | [Ref.] | [Ref.] | [Ref.] | [Ref.] | [Ref.] | [Ref.] | [Ref.] |
| mhow * age | -0.011 | -0.039, 0.017 | 0.444 | -0.016 | -0.042, 0.010 | 0.226 | -0.017 | -0.043, 0.008 | 0.188 |
| mho * age | -0.028 | -0.064, 0.007 | 0.120 | -0.028 | -0.061, 0.005 | 0.096 | -0.028 | -0.061, 0.005 | 0.095 |
| muhnw * age | -0.030 | -0.076, 0.015 | 0.191 | -0.037 | -0.079, 0.004 | 0.079 | -0.036 | -0.077, 0.006 | 0.089 |
| muhow * age | -0.011 | -0.045, 0.023 | 0.515 | -0.029 | -0.060, 0.002 | 0.070 | -0.028 | -0.059, 0.003 | 0.080 |
| muho * age | -0.054 | -0.092, -0.016 | 0.005 | -0.061 | -0.096, -0.026 | <0.001 | -0.059 | -0.094, -0.024 | **<0.001** |
| **Verbal fluency** |  |  |  |  |  |  |  |  |  |
| mhnw * age | [Ref.] | [Ref.] | [Ref.] | [Ref.] | [Ref.] | [Ref.] | [Ref.] | [Ref.] | [Ref.] |
| mhow * age | 0.009 | -0.014, 0.033 | 0.434 | 0.003 | -0.020, 0.025 | 0.821 | 0.002 | -0.020, 0.024 | 0.890 |
| mho * age | -0.017 | -0.047, 0.013 | 0.269 | -0.020 | -0.049, 0.008 | 0.158 | -0.021 | -0.049, 0.007 | 0.148 |
| muhnw * age | -0.022 | -0.060, 0.016 | 0.253 | -0.033 | -0.068, 0.003 | 0.070 | -0.032 | -0.067, 0.004 | 0.078 |
| muhow * age | -0.003 | -0.031, 0.025 | 0.829 | -0.019 | -0.046, 0.007 | 0.150 | -0.020 | -0.046, 0.007 | 0.145 |
| muho * age | -0.007 | -0.039, 0.024 | 0.652 | -0.019 | -0.049, 0.010 | 0.203 | -0.018 | -0.047, 0.012 | 0.245 |

CI = Confidence Interval. MHNW = Metabolically Healthy Normal Weight. MHO = Metabolically Healthy Obesity. MHOW = Metabolically Healthy Overweight. MUHNW = Metabolically Unhealthy Normal Weight. MUHO = Metabolically Unhealthy Obesity. MUHOW = Metabolically Unhealthy Overweight. TMT-B = Trail-Making Test part B.

**Model 1:** linear mixed‑model with random slopes and intercepts adjusted for age, sex, race, marital status, and education level at baseline.

**Model 2:** linear mixed‑model with random slopes and intercepts adjusted for age, sex, race, marital status, education level, alcohol use, smoker status, physical inactivity, and depressive symptoms at baseline.

Inverse probability weighting was used to adjust all models for attrition related to mortality and missing participation at each wave.

Supplementary Table 4. Associations between metabolic phenotypes and cognitive decline during eight years of follow-up using the last observation carried backward from wave 3 for missing cognitive performance information of participants < 55 years in wave 2 (n = 12,795).

|  | **Unadjusted** | | | **Model 1** | | | **Model 2** | | |
| --- | --- | --- | --- | --- | --- | --- | --- | --- | --- |
|  | **Beta** | **95% CI** | **p-value** | **Beta** | **95% CI** | **p-value** | **Beta** | **95% CI** | **p-value** |
| **Global Cognition** |  |  |  |  |  |  |  |  |  |
| mhnw * age | [Ref.] | [Ref.] | [Ref.] | [Ref.] | [Ref.] | [Ref.] | [Ref.] | [Ref.] | [Ref.] |
| mhow * age | 0.028 | 0.009, 0.048 | b | 0.017 | -0.001, 0.035 | 0.067 | 0.016 | -0.002, 0.034 | 0.081 |
| mho * age | 0.008 | -0.020, 0.037 | 0.561 | 0.000 | -0.026, 0.027 | 0.998 | 0.000 | -0.027, 0.026 | 0.981 |
| muho * age | -0.034 | -0.055, -0.013 | 0.001 | -0.041 | -0.060, -0.021 | **<0.001** | -0.040 | -0.060, -0.021 | **<0.001** |
| muhnw * age | -0.053 | -0.075, -0.030 | <0.001 | -0.053 | -0.074, -0.032 | **<0.001** | -0.053 | -0.074, -0.031 | **<0.001** |
| muhow * age | -0.026 | -0.045, -0.007 | 0.009 | -0.036 | -0.054, -0.018 | **<0.001** | -0.037 | -0.055, -0.019 | **<0.001** |
| **Memory** |  |  |  |  |  |  |  |  |  |
| mhnw * age | [Ref.] | [Ref.] | [Ref.] | [Ref.] | [Ref.] | [Ref.] | [Ref.] | [Ref.] | [Ref.] |
| mhow * age | 0.038 | 0.011, 0.065 | **0.005** | 0.024 | -0.002, 0.050 | 0.072 | 0.023 | -0.003, 0.049 | 0.089 |
| mho * age | 0.024 | -0.015, 0.063 | 0.225 | 0.013 | -0.026, 0.051 | 0.517 | 0.013 | -0.026, 0.051 | 0.519 |
| muhnw * age | -0.068 | -0.099, -0.037 | **<0.001** | -0.070 | -0.101, -0.040 | **<0.001** | -0.072 | -0.102, -0.041 | **<0.001** |
| muhow * age | -0.017 | -0.044, 0.010 | 0.218 | -0.030 | -0.057, -0.004 | **0.023** | -0.033 | -0.059, -0.007 | **0.014** |
| muho * age | -0.022 | -0.051, 0.007 | 0.135 | -0.033 | -0.061, -0.005 | **0.021** | -0.034 | -0.063, -0.006 | **0.017** |
| **TMT-B** |  |  |  |  |  |  |  |  |  |
| mhnw * age | [Ref.] | [Ref.] | [Ref.] | [Ref.] | [Ref.] | [Ref.] | [Ref.] | [Ref.] | [Ref.] |
| mhow * age | 0.010 | -0.022, 0.042 | 0.541 | 0.002 | -0.027, 0.031 | 0.885 | 0.002 | -0.027, 0.031 | 0.906 |
| mho * age | -0.012 | -0.058, 0.034 | 0.603 | -0.010 | -0.053, 0.032 | 0.639 | -0.010 | -0.052, 0.032 | 0.644 |
| muhnw * age | -0.033 | -0.070, 0.005 | 0.086 | -0.031 | -0.065, 0.003 | 0.077 | -0.027 | -0.061, 0.007 | 0.122 |
| muhow * age | -0.041 | -0.073, -0.009 | **0.011** | -0.055 | -0.085, -0.026 | **<0.001** | -0.053 | -0.083, -0.024 | **<0.001** |
| muho * age | -0.062 | -0.096, -0.028 | **<0.001** | -0.067 | -0.099, -0.035 | **<0.001** | -0.064 | -0.096, -0.032 | **<0.001** |
| **Verbal fluency** |  |  |  |  |  |  |  |  |  |
| mhnw * age | [Ref.] | [Ref.] | [Ref.] | [Ref.] | [Ref.] | [Ref.] | [Ref.] | [Ref.] | [Ref.] |
| mhow * age | 0.018 | -0.008, 0.045 | 0.180 | 0.007 | -0.018, 0.032 | 0.596 | 0.006 | -0.019, 0.032 | 0.626 |
| mho * age | -0.021 | -0.060, 0.019 | 0.303 | -0.025 | -0.062, 0.012 | 0.185 | -0.026 | -0.063, 0.011 | 0.172 |
| muhnw * age | -0.031 | -0.062, 0.000 | 0.050 | -0.035 | -0.064, -0.006 | **0.019** | -0.033 | -0.062, -0.004 | **0.026** |
| muhow * age | -0.024 | -0.051, 0.003 | 0.080 | -0.036 | -0.061, -0.011 | **0.005** | -0.035 | -0.060, -0.010 | **0.006** |
| muho * age | -0.030 | -0.059, -0.001 | **0.042** | -0.038 | -0.065, -0.011 | **0.006** | -0.036 | -0.063, -0.009 | **0.009** |

CI = Confidence Interval. MHNW = Metabolically Healthy Normal Weight. MHO = Metabolically Healthy Obesity. MHOW = Metabolically Healthy Overweight. MUHNW = Metabolically Unhealthy Normal Weight. MUHO = Metabolically Unhealthy Obesity. MUHOW = Metabolically Unhealthy Overweight. TMT-B = Trail-Making Test part B.

**Model 1:** linear mixed‑model with random slopes and intercepts adjusted for age, sex, race, marital status, and education level at baseline.

**Model 2:** linear mixed‑model with random slopes and intercepts adjusted for age, sex, race, marital status, education level, alcohol use, smoker status, physical inactivity, and depressive symptoms at baseline.

Inverse probability weighting was used to adjust all models for attrition related to mortality and missing participation at each wave.

Supplementary Table 5. Mediation by CRP levels of the association between metabolic phenotypes and cognitive decline (n = 12,795).

|  | **Total effect (95% CI)** | **Direct effect (95% CI)** | **Indirect effect (95% CI)** | **Proportion mediated** |
| --- | --- | --- | --- | --- |
| MHO*age | -0.000 (-0.025, 0.027) | 0.000 (-0.025, 0.028) | 0.000 (-0.007, 0.006) | 0% |
| MHOW*age | 0.016 (-0.002, 0.034) | 0.016 (-0.001, 0.034) | 0.000 (-0.004, 0.004) | 0% |
| MUHNW*age | -0.052 (-0.073, -0.032) | -0.052 (-0.072, -0.032) | 0.000 (-0.005, 0.004) | 0% |
| MUHOW*age | -0.037 (-0.057, -0.018) | -0.036 (-0.057, -0.018) | -0.001 (-0.005, 0.004) | 2.70% |
| MUHO*age | -0.040 (-0.059, -0.021) | -0.039 (-0.058, -0.020) | -0.001 (-0.007, 0.004) | 2.50% |

CI = Confidence Interval. MHNW = Metabolically Healthy Normal Weight. MHO = Metabolically Healthy Obesity. MHOW = Metabolically Healthy Overweight. MUHNW = Metabolically Unhealthy Normal Weight. MUHO = Metabolically Unhealthy Obesity. MUHOW = Metabolically Unhealthy Overweight.

Direct effect represents the association between metabolic obesity phenotypes and cognitive decline, independent of the mediator.

Indirect effect represents the portion of the association mediated by CRP levels.

Inverse probability weighting was used to adjust all models for attrition related to mortality and missing participation at each wave.

Supplementary Table 6. Random-effects variance components from the main linear mixed model

| **Random‐effects component** | **Variance** | **Std. Dev.** | **Correlation** |
| --- | --- | --- | --- |
| **Intercept** | 0.164 | 0.405 | — |
| **Slope** | 0.003 | 0.058 | -0.399 |
| **Residual** | 0.100 | 0.316 | — |

Supplementary Table 7. Associations between metabolic phenotypes and global cognitive decline during eight years of follow-up, using MHO as the reference (n = 12,795).

|  | **Unadjusted** | | | **Model 1** | | | **Model 2** | | |
| --- | --- | --- | --- | --- | --- | --- | --- | --- | --- |
|  | **Beta** | **95% CI** | **p-value** | **Beta** | **95% CI** | **p-value** | **Beta** | **95% CI** | **p-value** |
| MHO * age | [Ref.] | [Ref.] | [Ref.] | [Ref.] | [Ref.] | [Ref.] | [Ref.] | [Ref.] | [Ref.] |
| MHNW * age | -0.008 | -0.037, 0.020 | 0.561 | 0.000 | -0.027, 0.026 | 0.998 | 0.000 | -0.026, 0.027 | 0.981 |
| MHOW * age | 0.020 | -0.009, 0.049 | 0.175 | 0.017 | -0.010, 0.044 | 0.223 | 0.016 | -0.011, 0.044 | 0.236 |
| MUHNW * age | -0.061 | -0.092, -0.030 | **<0.001** | -0.053 | -0.082, -0.024 | **<0.001** | -0.052 | -0.081, -0.023 | **<0.001** |
| MUHOW * age | -0.034 | -0.063, -0.005 | **0.021** | -0.036 | -0.063, -0.009 | **0.009** | -0.036 | -0.064, -0.009 | **0.009** |
| MUHO * age | -0.042 | -0.072, -0.012 | **0.006** | -0.041 | -0.069, -0.012 | **0.005** | -0.040 | -0.068, -0.012 | **0.006** |

CI = Confidence Interval. MHNW = Metabolically Healthy Normal Weight. MHO = Metabolically Healthy Obesity. MHOW = Metabolically Healthy Overweight. MUHNW = Metabolically Unhealthy Normal Weight. MUHO = Metabolically Unhealthy Obesity. MUHOW = Metabolically Unhealthy Overweight. TMT-B = Trail-Making Test part B.

**Model 1:** linear mixed‑model with random slopes and intercepts adjusted for age, sex, race, marital status, and education level at baseline.

**Model 2:** linear mixed‑model with random slopes and intercepts adjusted for age, sex, race, marital status, education level, alcohol use, smoker status, physical inactivity, and depressive symptoms at baseline. Inverse probability weighting was used to adjust for attrition related to mortality and missing participation at each wave.

Inverse probability weighting was used to adjust all models for attrition related to mortality and missing participation at each wave.

Supplementary Table 8. Associations between metabolic phenotypes and global cognitive decline during eight years of follow-up, using MUHO as the reference (n = 12,795).

|  | **Unadjusted** | | | **Model 1** | | | **Model 2** | | |
| --- | --- | --- | --- | --- | --- | --- | --- | --- | --- |
|  | **Beta** | **95% CI** | **p-value** | **Beta** | **95% CI** | **p-value** | **Beta** | **95% CI** | **p-value** |
| MUHO * age | [Ref.] | [Ref.] | [Ref.] | [Ref.] | [Ref.] | [Ref.] | [Ref.] | [Ref.] | [Ref.] |
| MHNW * age | 0.034 | 0.013, 0.055 | **0.002** | 0.041 | 0.021, 0.060 | **<0.001** | 0.040 | 0.021, 0.060 | **<0.001** |
| MHOW * age | 0.062 | 0.040, 0.084 | **<0.001** | 0.058 | 0.037, 0.078 | **<0.001** | 0.056 | 0.036, 0.077 | **<0.001** |
| MHO * age | 0.042 | 0.012, 0.072 | **0.006** | 0.041 | 0.012, 0.069 | **0.005** | 0.040 | 0.012, 0.068 | **0.006** |
| MUHNW * age | -0.019 | -0.044, 0.006 | 0.140 | -0.012 | -0.036, 0.011 | 0.300 | -0.012 | -0.036, 0.011 | 0.292 |
| MUHOW * age | 0.008 | -0.014, 0.030 | 0.485 | 0.005 | -0.016, 0.025 | 0.666 | 0.003 | -0.017, 0.024 | 0.746 |

CI = Confidence Interval. MHNW = Metabolically Healthy Normal Weight. MHO = Metabolically Healthy Obesity. MHOW = Metabolically Healthy Overweight. MUHNW = Metabolically Unhealthy Normal Weight. MUHO = Metabolically Unhealthy Obesity. MUHOW = Metabolically Unhealthy Overweight. TMT-B = Trail-Making Test part B.

**Model 1:** linear mixed‑model with random slopes and intercepts adjusted for age, sex, race, marital status, and education level at baseline.

**Model 2:** linear mixed‑model with random slopes and intercepts adjusted for age, sex, race, marital status, education level, alcohol use, smoker status, physical inactivity, and depressive symptoms at baseline. Inverse probability weighting was used to adjust for attrition related to mortality and missing participation at each wave.

Inverse probability weighting was used to adjust all models for attrition related to mortality and missing participation at each wave.

Supplementary Table 9. Association between body mass index and global cognitive decline during eight years of follow-up (n = 12,795).

|  | **Unadjusted** | | | **Model 1** | | | **Model 2** | | | **Model 3** | | |
| --- | --- | --- | --- | --- | --- | --- | --- | --- | --- | --- | --- | --- |
|  | **Beta** | **95% CI** | **p-value** | **Beta** | **95% CI** | **p-value** | **Beta** | **95% CI** | **p-value** | **Beta** | **95% CI** | **p-value** |
| BMI * age | 0.000 | -0.002, 0.001 | 0.800 | -0.001 | -0.002, 0.000 | 0.186 | -0.001 | -0.002, 0.000 | 0.170 | -0.001 | -0.002, 0.001 | 0.300 |

CI = Confidence Interval. BMI = Body Mass Index.

**Model 1:** linear mixed‑model with random slopes and intercepts adjusted for age, sex, race, marital status, and education level at baseline.

**Model 2:** linear mixed‑model with random slopes and intercepts adjusted for age, sex, race, marital status, education level, alcohol use, smoker status, physical inactivity, and depressive symptoms at baseline.

**Model 3:** linear mixed-model with random slopes and intercepts adjusted for age, sex, race, marital status, education level, alcohol use, smoker status, physical inactivity, depressive symptoms, BMI, hypertension, diabetes, dyslipidemia, and cardiovascular disease at baseline.

Inverse probability weighting was used to adjust all models for attrition related to mortality and missing participation at each wave.

Supplementary Table 10. Association between WHR and global cognitive decline during eight years of follow-up (n = 12,795).

|  | **Unadjusted** | | | **Model 1** | | | **Model 2** | | | **Model 3** | | |
| --- | --- | --- | --- | --- | --- | --- | --- | --- | --- | --- | --- | --- |
|  | **Beta** | **95% CI** | **p-value** | **Beta** | **95% CI** | **p-value** | **Beta** | **95% CI** | **p-value** | **Beta** | **95% CI** | **p-value** |
| WHR * age | -0.016 | -0.022, -0.010 | <0.001 | -0.020 | -0.026, -0.014 | <0.001 | -0.020 | -0.026, -0.014 | <0.001 | -0.019 | -0.025, -0.013 | <0.001 |

CI = Confidence Interval. WHR = Waist-to-Hip Ratio

**Model 1:** linear mixed‑model with random slopes and intercepts adjusted for age, sex, race, marital status, and education level at baseline.

**Model 2:** linear mixed‑model with random slopes and intercepts adjusted for age, sex, race, marital status, education level, alcohol use, smoker status, physical inactivity, and depressive symptoms at baseline.

**Model 3:** linear mixed-model with random slopes and intercepts adjusted for age, sex, race, marital status, education level, alcohol use, smoker status, physical inactivity, depressive symptoms, BMI, hypertension, diabetes, dyslipidemia, and cardiovascular disease at baseline.

Inverse probability weighting was used to adjust all models for attrition related to mortality and missing participation at each wave.

Supplementary Table 11. Associations between metabolic phenotypes and cognitive decline during eight years of follow-up stratified by race (n = 12,355).

|  | **White (n = 6,767)** | | | **Black/Brown (n = 5,588)** | | |
| --- | --- | --- | --- | --- | --- | --- |
|  | **Beta** | **95% CI** | **p-value** | **Beta** | **95% CI** | **p-value** |
| MHNW * age | [Ref.] | [Ref.] | [Ref.] | [Ref.] | [Ref.] | [Ref.] |
| MHOW * age | 0.020 | -0.002, 0.043 | 0.078 | 0.019 | -0.013, 0.050 | 0.247 |
| MHO * age | -0.017 | -0.050, 0.017 | 0.329 | 0.027 | -0.017, 0.071 | 0.225 |
| MUHNW * age | -0.052 | -0.079, -0.024 | **<0.001** | -0.038 | -0.073, -0.003 | **0.035** |
| MUHOW * age | -0.054 | -0.077, -0.030 | **<0.001** | -0.007 | -0.038, 0.023 | 0.643 |
| MUHO * age | -0.034 | -0.060, -0.008 | **0.009** | -0.028 | -0.060, 0.004 | 0.082 |

CI = Confidence Interval. MHNW = Metabolically Healthy Normal Weight. MHO = Metabolically Healthy Obesity. MHOW = Metabolically Healthy Overweight. MUHNW = Metabolically Unhealthy Normal Weight. MUHO = Metabolically Unhealthy Obesity. MUHOW = Metabolically Unhealthy Overweight.

Linear mixed‑model with random slopes and intercepts adjusted for age, sex, race, marital status, education level, alcohol use, smoker status, physical inactivity, and depressive symptoms at baseline.

Inverse probability weighting was used to adjust all models for attrition related to mortality and missing participation at each wave.

Supplementary Table 12. Associations between metabolic phenotypes and cognitive decline during eight years of follow-up stratified by sex (n = 12,795).

|  | **Men (5,740)** | | | **Women (n = 7,055)** | | |
| --- | --- | --- | --- | --- | --- | --- |
|  | **Beta** | **95% CI** | **p-value** | **Beta** | **95% CI** | **p-value** |
| MHNW * age | [Ref.] | [Ref.] | [Ref.] | [Ref.] | [Ref.] | [Ref.] |
| MHOW * age | 0.042 | 0.012, 0.072 | **0.006** | 0.001 | -0.022, 0.023 | 0.945 |
| MHO * age | 0.031 | -0.019, 0.080 | 0.224 | -0.015 | -0.046, 0.015 | 0.328 |
| MUHNW * age | -0.039 | -0.072, -0.007 | **0.018** | -0.054 | -0.082, -0.026 | **<0.001** |
| MUHOW * age | -0.018 | -0.045, 0.010 | 0.215 | -0.044 | -0.069, -0.019 | **<0.001** |
| MUHO * age | -0.034 | -0.066, -0.002 | **0.035** | -0.040 | -0.065, -0.015 | **0.002** |

CI = Confidence Interval. MHNW = Metabolically Healthy Normal Weight. MHO = Metabolically Healthy Obesity. MHOW = Metabolically Healthy Overweight. MUHNW = Metabolically Unhealthy Normal Weight. MUHO = Metabolically Unhealthy Obesity. MUHOW = Metabolically Unhealthy Overweight.

Linear mixed‑model with random slopes and intercepts adjusted for age, sex, race, marital status, education level, alcohol use, smoker status, physical inactivity, and depressive symptoms at baseline.

Inverse probability weighting was used to adjust all models for attrition related to mortality and missing participation at each wave.
